# Antioxidative stress protein SRXN1 can be used as a radiotherapy prognostic marker for prostate cancer

**DOI:** 10.1186/s12894-023-01319-1

**Published:** 2023-09-19

**Authors:** Xing Wang, Jiandi Yu, Huali Wen, Junfeng Yan, Kun Peng, Haiyong Zhou

**Affiliations:** Department of Urology, Zhejiang Hospital, # 1229, Gudun Road, Hangzhou, 310030 China

**Keywords:** Prostate cancer, Radiotherapy, SRXN1, Antioxidant stress, Prognostic marker

## Abstract

**Purpose:**

To explore the mechanisms of radiotherapy resistance and search for prognostic biomarkers for prostate cancer.

**Methods:**

The GSE192817 and TCGA PRAD datasets were selected and downloaded from the GEO and UCSC Xena databases. Differential expression and functional annotation analyses were applied to 52 tumour cell samples from GSE192817. Then, the ssGSEA or GSVA algorithms were applied to quantitatively score the biological functional activity of samples in the GSE192817 and TCGA PRAD datasets, combined with specific gene sets collected from the Molecular Signatures Database (MSigDB). Subsequently, the Wilcoxon rank-sum test was used to compare the differences in ssGSEA or GSVA scores among cell types or PRAD patients. Moreover, radiotherapy resistance-associated gene screening was performed on DU145 and PC3 cells (prostate cancer cells), and survival analysis was used to evaluate the efficacy of these genes for predicting the prognosis of PRAD patients.

**Results:**

A total of 114 genes that were differentially expressed in more than two different cancer cell types and associated with either sham surgery or radiotherapy treatment (X-ray or photon irradiation) were detected in cancer cells from GSE192817. Comparison of DNA damage-related ssGSEA scores between sham surgery and radiotherapy treatment in prostate cancer cells (DU145 and PC3) showed that photon irradiation was potentially more effective than X-ray treatment. In the TCGA PRAD dataset, patients treated with radiotherapy had much higher “GOBP_CELLULAR_RESPONSE_TO_DNA_DAMAGE_STIMULUS”, “GOBP_G2_DNA_DAMAGE_CHECKPOINT” and “GOBP_INTRA_S_DNA_DAMAGE_CHECKPOINT” GSVA scores, and the Wilcoxon rank-sum test p values were 0.0005, 0.0062 and 0.0800, respectively. Furthermore, SRXN1 was upregulated in DU145 cells (resistant to X-ray irradiation compared to PC3 cells) after radiotherapy treatment, and low SRXN1 expression in patients was beneficial to radiotherapy outcomes. The log-rank test *p* value for PFS was 0.0072.

**Conclusions:**

Radiotherapy can damage DNA and induce oxidative stress to kill tumour cells. In this study, we found that SRXN1, as an antioxidative stress gene, plays an important role in radiotherapy for prostate cancer treatment, and this gene is also a potential biomarker for predicting the prognosis of patients treated with radiotherapy.

## Introduction

Prostate cancer ranks second among all male malignancies in the world [1]. In recent years, the cancer spectrum in China has shifted from a developing country to a developed country, and the incidence of prostate cancer is increasing rapidly [2]. Currently, the main causes of prostate cancer are genetic factors (BRCA1 or BRCA2 mutation) [3], environmental factors (serum As and Zn levels) [4], dietary factors (red meat, high fat) [5], age, family history [5], etc. Prostate cancer may be asymptomatic in the early stage, and the clinical symptoms of advanced patients are mainly urinary retention and back pain [6]. At present, serum prostate-specific antigen (PSA) is often used clinically to screen for prostate cancer because it can be used to detect early prostate cancer and promote the treatment of patients in a timely manner [7]. For localized prostate cancer, radical prostatectomy, radiotherapy techniques (external beam, proton beam, brachytherapy), and androgen deprivation therapy (ADT) are the main treatments [8, 9]. Seok-Joo Chun and his colleagues showed similar oncological prostate cancer-specific survival (PCSS), overall survival (OS), and distant metastasis-free survival (DMFS) outcomes between high-risk prostate cancer patients receiving external beam radiotherapy (EBRT) or radical prostatectomy (RP) [10].

Ionizing radiation is an important way to treat malignant diseases [11]. Radiation therapy (RT), including external beam radiotherapy (EBRT) and internal radioisotope therapy (RIT), is an important type of ionizing radiation that has been widely used in clinical tumour treatment [12]. RT is the use of high-energy ionizing beams to directly or indirectly ionize target cells, promote DNA damage, and achieve the purpose of controlling or killing malignant tumour cells [13]. Because of its significant efficacy, many cancer patients incorporate radiotherapy into their first-line cancer treatment regimens. Radiotherapy is minimally invasive [14], and the dose can be flexibly adjusted according to the actual situation of each patient [15]. Using X-ray irradiation to treat tumours has a certain curative effect, but due to the low attenuation coefficient of X-rays in organisms, the excessive ionizing radiation produced has great lethality towards normal cells [16, 17]. Proton beam therapy (PBT) is a new type of radiation therapy that can improve the survival of patients and reduce the adverse reactions caused by radiation by improving the cure rate of local tumours and reducing damage to normal organs [18]. PBT has been shown to have good efficacy in the treatment of various cancers, such as head and neck cancer [19], glioblastoma [20], and prostate cancer [21].

In this study, according to the differential expression analysis and quantitative scoring based on the ssGSVA algorithm in GSE192817, radiotherapy was found to significantly enhance DNA damage and cause tumour cell death. According to the analysis of prostate cancer cells, eight genes related to radiotherapy resistance were screened, and SRXN1 was identified as a potential biomarker for predicting the prognosis of prostate cancer in patients treated with radiotherapy.

## Methods

### Data collection and pretreatment

With “radiotherapy” and “cancer” as the keywords, we searched and selected the GSE192817 dataset from the GEO database (https://www.ncbi.nlm.nih.gov/geo/query/acc.cgi?acc = GSE192817). A total of 52 cell samples from GSE192817 were treated with sham surgery, proton irradiation or X-ray irradiation, and the majority of samples were collected after approximately 7 days. Combined with the sequencing platform GPL20844 of this dataset and the R package "biomaRt" (Version: 2.48.3), the microarray probe IDs were converted into gene symbols, and the gene types were classified. Moreover, TCGA prostate adenocarcinoma (PRAD) was downloaded from the UCSC Xena database (https://tcga.xenahubs.net), which included gene expression data and clinical follow-up data for patients.

### Differentially expressed gene screening and functional annotation

Differences in protein-coding genes were compared between the radiotherapy group (X-ray radiotherapy and proton radiotherapy) and the control group (sham surgery) based on the Wilcoxon rank sum test. Potentially differentially expressed genes in six types of cells were obtained based on a *p*-value test result of < 0.1, and genes that were differentially expressed in at least two types of cells were retained. In addition, the DAVID tool (https://david.ncifcrf.gov/) was used to perform GO functional annotation on the above differentially expressed genes.

### Quantitative scoring of DNA damage-related functions

A total of four gene sets, namely, "GOBP_CELLULAR_RESPONSE_TO_, DNA_DAMAGE_STIMULUS", "GOBP_G2_DNA_DAMAGE_CHECKPOINT", "GOBP_INTRA_S_DNA_DAMAGE_CHECKPOINT" and "GOBP_INTRINSIC_, APOPTOTIC_SIGNALING_PATHWAY_IN_RESAMAGE_TO_TO", were downloaded from the MSigDB (http://software.broadinstitute.org/gsea/msigdb). Subsequently, the "ssGSEA" algorithm in the R package "GSVA" (Version: 1.42.0) was used to quantitatively score the samples from the six types of cells in the GSE192817 dataset after 420 h of radiotherapy. For patients in the TCGA PRAD dataset, we used the "GSVA" algorithm for quantitative scoring and applied the Wilcoxon rank sum test for different comparisons.

### Screening and survival analysis of X-ray radiotherapy tolerance genes in prostate cancer cells

The Wilcoxon rank-sum test was used to compare the differences in genes between two prostate cancer cell types (DU145 and PC3) in the TCGA PRAD dataset according to either X-ray radiotherapy or sham surgery treatment, and FC values were calculated. Differentially expressed genes with a *p*-value test result of < 0.1 between the two types of cells were retained. The R package "survival" (Version: 3.2–11) was used to perform survival analysis on the above candidate genes, a univariate Cox proportional hazards model was constructed, and the log-rank test was performed. Finally, the HR [95% CI] of the risk model and the *p*-value test results were recorded.

### Correlation analysis and survival analysis of SRXN1 and the oxidative stress response

The gene set "GOBP_RESPONSE_TO_OXIDATIVE_STRESS" was identified and downloaded from the MSigDB, and a correlation analysis was performed between the gene expression of SRXN1 in the TCGA-PRAD dataset and the GSVA of the patient's oxidative stress response; the Pearson correlation coefficient (PCC) was calculated, and a correlation test (cor.test) was performed. The Wilcoxon rank sum test was used to compare the differences between the SRXN1 high-expression group and the SRXN1 low-expression group. The R package "survival" (Version: 3.2–11) was used to evaluate the oxidation. Survival analysis was performed on the GSVA score of the stress response, a univariate Cox proportional hazards model was constructed, the log-rank test was performed, and the HR [95% CI] of the risk model and the *p*-value test results were finally recorded.

## Results

### Differentially expressed genes identified in tumour cells after radiotherapy

**Fig. 1:**
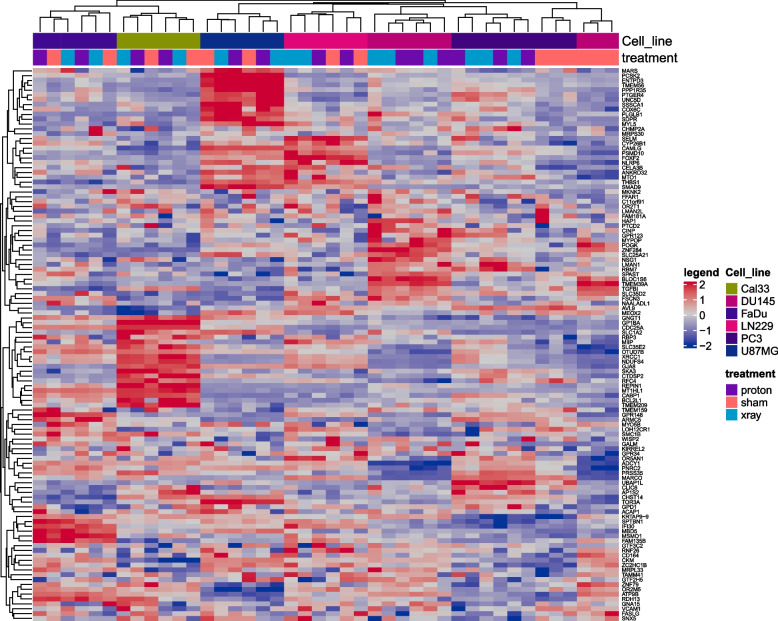
Gene expression heatmap of 114 differentially expressed genes after radiotherapy in the GSE192817 dataset

To explore the intrinsic molecular mechanism of radiotherapy resistance in tumour cells, with “radiotherapy” and “cancer” as the keywords, we searched and selected the GSE192817 dataset from the GEO database. In GSE192817, we analysed 52 cell samples (Table 1), including 16 DU145 (3 with proton irradiation, 3 with X-ray irradiation and 3 with sham surgery; 1 with proton irradiation, 1 with X-ray irradiation and 1 with sham surgery collected after 4 h of irradiation; 1 with proton irradiation, 1 with X-ray irradiation and 1 with sham surgery collected after 12 h of irradiation), 12 PC3 (3 with proton irradiation, 3 with X-ray irradiation and 3 with sham surgery collected after 420 h of irradiation; 1 with proton irradiation, 1 with X-ray irradiation and 1 with sham surgery collected after 4 h of irradiation), 6 FaDu (2 with proton irradiation, 2 with X-ray irradiation and 2 with sham surgery collected after 420 h of irradiation), 6 Cal33 (2 with proton irradiation, 2 with X-ray irradiation and 2 with sham surgery collected after 420 h of irradiation), 6 LN229 (2 with proton irradiation, 2 with X-ray irradiation and 2 with sham surgery collected after 420 h of irradiation), and 6 U87MG (2 with proton irradiation, 2 with X-ray irradiation and 2 with sham surgery collected after 420 h of irradiation). In addition, according to the mapping relationship between probe ID and gene name, 34,729 genes and 18,465 protein-coding genes (PCGs) were screened from 62,976 probes, and the type of each gene was obtained. The Wilcoxon rank sum test showed that there were 955, 907, 24, 32, 27 and 36 differentially expressed genes in the six cell types (DU145, PC3, FaDu, cal33, ln229 and U87MG), and we screened 114 genes that were differentially expressed in at least two cell types (Fig. 1).

**Table 1:** Details of the GSE192817 dataset cells

Collected after 420 h	Sham surgery	Proton irradiation	X-ray irradiation
DU145	3	3	3
PC3	3	3	3
FaDu	2	2	2
Cal33	2	2	2
LN229	2	2	2
U87MG	2	2	2
Collected after 12 h			
DU145	1	1	1
Collected after 4 h			
DU145	1	1	1
PC3	1	1	1

**Fig. 2:**
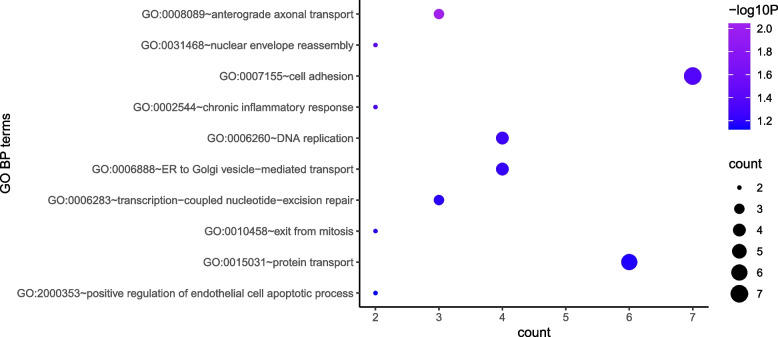
Functional annotation results of 114 differentially expressed genes after radiotherapy in cancer cells

GO functional annotation results showed that 114 differentially expressed genes were mainly enriched in 4 GO items, including "GO:0007155 ~ cell adhesion", "GO:0031468 ~ nuclear envelope reassembly", "GO:0002544 ~ chronic inflammatory response", and "GO:0006260 ~ DNA replication" (Table 2, Fig. 2).

**Table 2:** Functional annotation results of 114 differentially expressed genes after radiotherapy in cancer cells

Term	Count	*P* Value
GO:0008089 ~ anterograde axonal transport	3	0.009045335053033
GO:0031468 ~ nuclear envelope reassembly	2	0.038136934988615
GO:0007155 ~ cell adhesion	7	0.042066465470811
GO:0002544 ~ chronic inflammatory response	2	0.048766437520776
GO:0006260 ~ DNA replication	4	0.054865474777133
GO:0006888 ~ ER to Golgi vesicle-mediated transport	4	0.059225950918285
GO:0006283 ~ transcription-coupled nucleotide-excision repair	3	0.063403316447113
GO:0010458 ~ exit from mitosis	2	0.064493178030768
GO:0015031 ~ protein transport	6	0.06831494368806
GO:2000353 ~ positive regulation of endothelial cell apoptotic process	2	0.074834491043404
GO:0090201 ~ negative regulation of release of cytochrome c from mitochondria	2	0.090134781767891
GO:0030198 ~ extracellular matrix organization	4	0.095099288222236
GO:0042462 ~ eye photoreceptor cell development	2	0.095179041992072

### Effects of different tumour-derived cells on responses to DNA damage stimuli

**Fig. 3:**
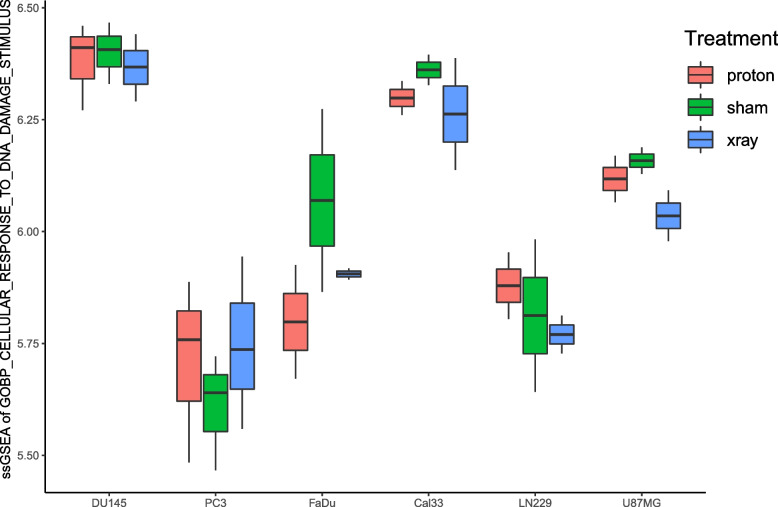
Differences in ssGSEA scores in response to DNA damage stimulation between different treatments of different cell types

A total of 869 related genes were obtained from the gene set in response to DNA damage stimuli. The ssGSEA results showed that PC3 cells were very sensitive to radiotherapy, and the DNA was significantly damaged after treatment. In contrast, FaDu cells of head and neck cancer were extremely resistant to radiotherapy (Fig. 3). This suggests that cells of different cancer types respond differently to radiotherapy. In addition, except for FaDu cells, the scores of the other cell types treated with proton radiotherapy were higher than those treated with X-ray radiotherapy (Fig. 3), indicating that proton radiotherapy causes more DNA damage to tumour cells and is more conducive to killing tumour cells.

**Fig. 4:**
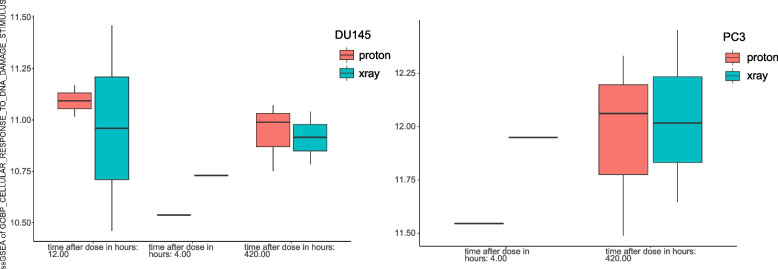
Differences in ssGSEA scores in response to DNA damage stimulation between different radiotherapy times in two prostate cancer cell types

Furthermore, the ssGSEA results showed significantly stronger DNA damage after 420 h of radiation in two prostate cancer cell types (DU145 and PC3) than after 4 h of radiation (only one sample was sequenced), but the DU145 ssGSEA score after 12 h of radiation was slightly higher than that after 420 h of radiotherapy (Fig. 4), indicating that the timing of radiotherapy may also affect the stimulation of DNA damage to tumour cells. For both cell types, proton radiotherapy was performed at different time points. Stimulation was slightly stronger than X-ray radiotherapy, suggesting that proton radiotherapy may be superior to X-ray radiotherapy in these two prostate cancer cell types (DU145 and PC3).

**Fig. 5:**
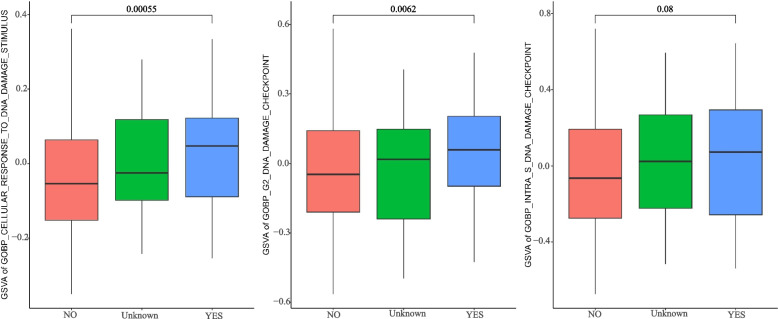
Differences in GSVA scores for three DNA damage-related biological functions among patients in the TCGA PRAD dataset

After that, we selected patients from the TCGA PRAD dataset and designated them as “Yes”, “No” and “Unknown” (without clear information) based on radiotherapy treatment, and the GSVA algorithm was used for quantitative scoring. The p values of the “GOBP_CELLULAR_RESPONSE_TO_DNA_DAMAGE_STIMULUS”, “GOBP_G2_DNA_DAMAGE_CHECKPOINT” and “GOBP_INTRA_S_DNA_DAMAGE_CHECKPOINT” GSVA scores between patients treated with and without radiotherapy were 0.0005, 0.0062 and 0.0800, respectively (Fig. 5). The boxplot shows that the median values of GSVA scores in patients treated with radiotherapy were higher than those in patients treated without radiotherapy, which indicated that DNA damage was more intense in radiotherapy-treated patients. Unknown patients are those patients without clear radiotherapy treatment information.

### Analysis of the intrinsic mechanism of PC3 cell sensitivity to radiotherapy

**Fig. 6:**
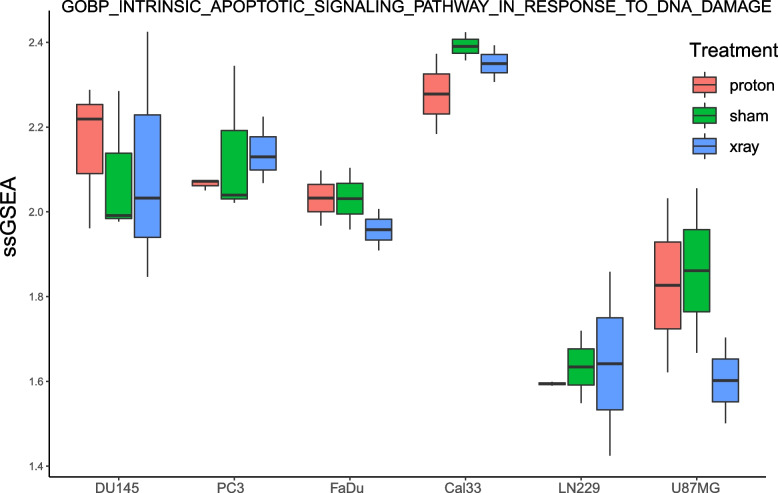
Differences in GSVA scores of apoptosis signalling pathways in GSE192817 dataset cells

One hundred related genes were obtained from the DNA damage response endogenous apoptosis signalling pathway gene set. Combined with the expression data of the cells in the GSE192817 dataset, the ssGSEA results showed that DU145 cells were more sensitive to proton radiotherapy than PC3 cells and that PC3 cells were more sensitive to X-ray radiotherapy (Fig. 6).

The Wilcoxon rank sum test revealed 8 genes that were differentially expressed in the two cell types after radiotherapy. The differences in FC values are shown in Table 3. The results showed that SRXN1, FSCB, GALR3, TOR1B, RSPO4 and APOL5 were upregulated in DU145 cells after radiotherapy but downregulated in PC3 cells. The gene expression of FSCB and APOL5 was not detected in most of the samples, and these genes were excluded from the analysis; therefore, there was a possible association between genes differentially expressed in both cell types and the sensitivity of PC3 cells to X-ray radiotherapy.

**Table 3:** FC values of 8 genes differentially expressed in both DU145 and PC3 cells after radiotherapy

Gene	FC for DU145	Wilcox.test *p* value	FC for PC3	Wilcox.test *p* value
SRXN1	1.1176	0.0765	0.9452	0.0765
FSCB	1.1035	0.0765	0.9446	0.0765
GALR3	1.0780	0.0722	0.9666	0.0765
TOR1B	1.0718	0.0765	0.9551	0.0765
CDH16	1.0653	0.0765	1.0676	0.0765
RSPO4	1.0572	0.0765	0.9490	0.0593
APOL5	1.0500	0.0722	0.9055	0.0765
WFDC5	0.9599	0.0765	0.9534	0.0722

**Fig. 7:**
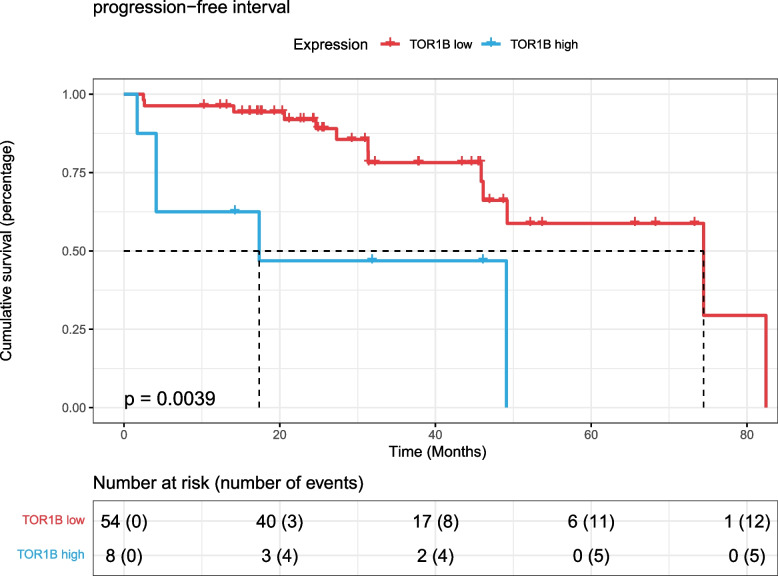
K‒M survival curve of progression-free interval survival with TOR1B in TCGA PARD dataset radiotherapy patients

According to the gene expression of TOR1B, 62 patients treated with radiotherapy were divided into a high-expression group (expression value higher than 80.9652) and a low-expression group (expression value lower than 80.9652). The HR [95% CI] was 4.326 [1.472–12.72], and the log-rank test* p* value was 0.0039 (Fig. 7). This indicates that patients with prostate cancer with high expression of TOR1B are not suitable for radiotherapy.

**Fig. 8:**
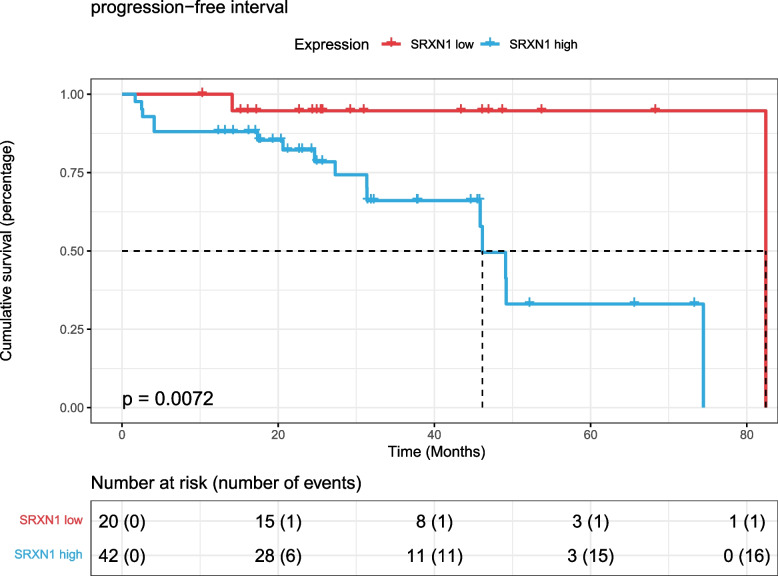
K‒M survival curves of progression-free interval survival for SRXN1 in TCGA PARD dataset radiotherapy patients

Similarly, according to the gene expression of SRXN1, 62 patients treated with radiotherapy were divided into two groups with high and low expression, with 71.4246 as the threshold. The HR [95% CI] of the final constructed univariate Cox proportional hazards model was 9.653 [1.275–73.09], and the log-rank test *p* value was 0.0072 (Fig. 8). This shows that prostate cancer patients with high expression of SRXN1 are not suitable for radiotherapy.

**Fig. 9:**
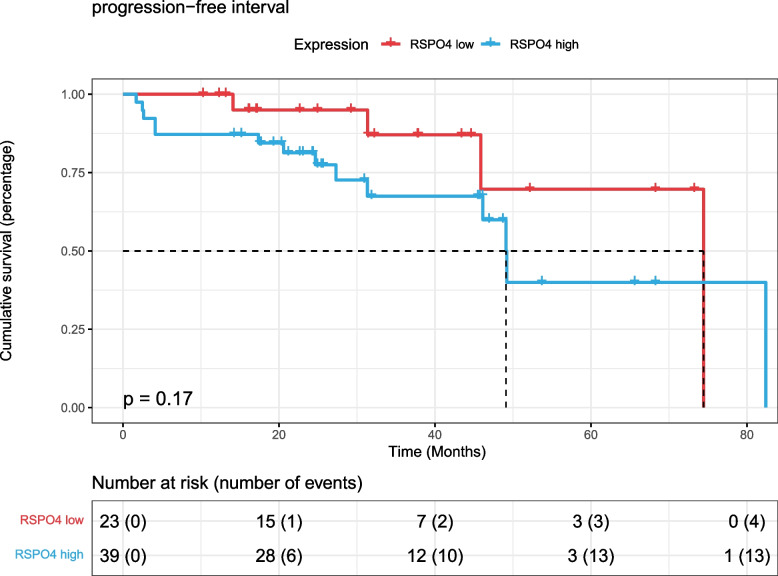
K‒M survival curve of progression-free interval survival with RSPO4 in TCGA PARD dataset radiotherapy patients

**Fig. 10:**
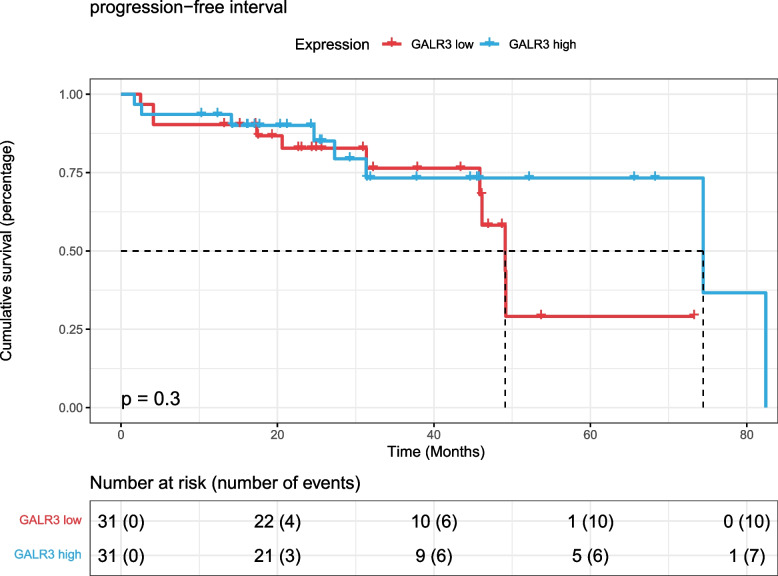
K‒M survival curve of progression-free interval survival with GALR3 in TCGA PARD dataset radiotherapy patients

Finally, according to the gene expression of RSPO4, 62 patients treated with radiotherapy were divided into two groups with high and low expression with 8.1409 as the threshold. The HR [95% CI] of the final constructed univariate Cox proportional hazards model was 2.152 [0.6982–6.635], and the log-rank test *p* value was 0.17 (Fig. 9). According to the gene expression of GALR3, the patients were divided into two groups with high and low expression with 4.1396 as the threshold. The HR [95% CI] of the final constructed univariate Cox proportional hazards model was 0.5858 [0.2115–1.623], and the log-rank test *p* value was 0.17 (Fig. 10). The above survival analysis results show that RSPO4 and GALR3 cannot be effectively used to predict the prognosis of prostate cancer patients undergoing radiotherapy.

### SRXN1 affects patient radiotherapy response through antioxidative stress

**Fig. 11:**
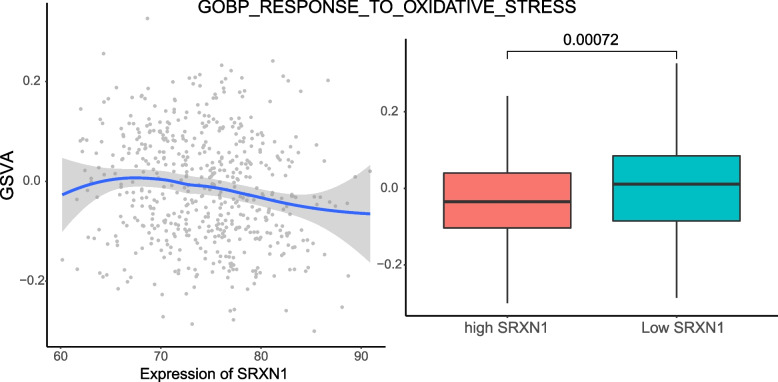
Correlation of SRXN1 expression in patients with GSVA scores in response to oxidative stress in the TCGA PRAD dataset

A total of 444 related genes were obtained from the "GOBP_RESPONSE_TO_OXIDATIVE_STRESS" gene set, and GSVA revealed functional quantification scores for TCGA PRAD patients. Correlation analysis showed that the gene expression of SRXN1 in patients was significantly negatively correlated with GSVA in response to oxidative stress. The PCC was -0.1536, and the correlation test p value was 0.0003, which indicates that SRXN1 plays an antioxidative stress role (Fig. 11). In addition, we divided TCGA PRAD patients into two groups with high and low expression according to the mean expression of SRXN1 (74.44408). The Wilcoxon rank sum test was used to compare the differences. The results showed that the GSVA score of the oxidative stress response in patients with low SRXN1 expression was significantly higher than that in patients with high SRXN1 expression (*p* value of 0.0072) (Fig. 11).

**Fig. 12:**
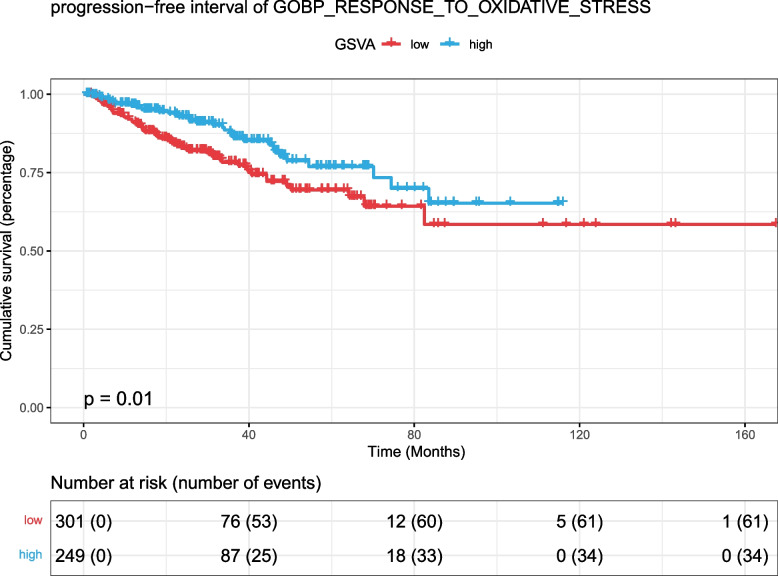
K‒M survival curves for progression-free interval survival by GSVA score in response to oxidative stress in the TCGA PARD dataset

Using the GSVA score of the oxidative stress response, 550 patients in the TCGA PRAD dataset were divided into two groups with 0 as the threshold. The results of survival analysis showed that the HR [95% CI] of the constructed univariate Cox proportional hazards model was 0.5796 [0.3806–0.8828], with a log-rank test *p* value of 0.01 (Fig. 12). This finding shows that ROS-sensitive tumour cells are also sensitive to radiotherapy, and the important regulatory role of SRXN1 may be used as an important biological marker for adjuvant radiotherapy in the future.

## Discussion

DNA damage refers to physical or chemical changes in DNA within cells, including exogenous and endogenous stress factors such as chemical substances, radiation, and free radicals [22]. The most common form of DNA damage is DNA single-strand breaks, but DNA double-strand breaks are the most serious type of DNA damage and are more lethal to cells [23]. It has been reported that more than half of cancer patients will use radiotherapy as part of their whole cancer treatment. DNA double-strand breaks induced by ionizing radiation during radiotherapy are the main cause of cell death [24]. Radiation therapy is the main treatment for many cancers, and neoadjuvant or adjuvant radiation therapy can also be used before or after other treatments, such as chemotherapy and surgery [25]. Studies have shown that the DNA damage response pathway plays an important role in the progression of prostate cancer and that mutations in DNA damage response-related genes can lead to a more aggressive prostate cancer phenotype, leading to an increased likelihood of distant metastasis of prostate cancer [26]. This study found that the PC3 prostate cancer cells were very sensitive to radiotherapy and that the DNA of PC3 cells was significantly damaged after radiotherapy. In addition, Francesco Marampon et al. found that downregulating cyclin D1 in the NHEJ and HR pathways of DNA double-strand break repair can increase radiation-induced DNA damage and significantly increase the radiosensitivity of prostate cancer cells [27]. Proton radiation therapy uses a beam of protons instead of traditional photons or X-ray beams to more precisely treat tumours. In vitro studies show that proton beam irradiation causes more DNA damage than other types of irradiation [28]. Potential differences in DNA damage between proton beam irradiation and gamma, photon, or X-ray irradiation may be due to different energy release trajectories (Bragg peaks) and microscopic patterns of energy deposition [29]. This study found that after radiotherapy at different time points, the stimulation of DNA damage by proton radiotherapy was slightly higher than that of X-ray radiotherapy.

Torsin 1B (TOR1B) mainly exists in the endoplasmic reticulum and nuclear membrane, and it plays an important role in maintaining the integrity of the nuclear membrane and endoplasmic reticulum [30]. In recent years, studies have found that TOR1B plays an important role in the development of malignant tumours. Liu Weiling et al. [31] found that the expression level of TOR1B was upregulated in KYSE150R and KYSE450R oesophageal squamous cell carcinoma cells, but there are few studies on this gene in prostate cancer. This study found that prostate cancer patients with high expression of TOR1B had a poor prognosis after radiotherapy. Sulfiredoxin 1 (SRXN1) is a key factor in the antioxidant response in eukaryotic cells [32]. SRXN1 is an antioxidant enzyme that protects host cells from oxidative damage by catalysing the reduction of peroxides to reductants [33]. Studies have shown that SRXN1 is overexpressed in a variety of malignant tumours, including breast cancer [34], colorectal cancer, lung cancer, prostate cancer, and skin cancer [35]. In addition, Caroline N. Barquilha et al. [36] found that SRXN1 overexpression in patients with advanced prostate cancer was associated with poor prognosis and identified SRXN1 as a potential therapeutic target for prostate cancer, which is consistent with our results. This study shows that the expression of SRXN1 is closely related to the prognosis of patients and that the overexpression of SRXN1 is not conducive to the prognosis of patients. We speculate that the high expression of SRXN1 will produce oxidative stress resistance in prostate cancer cells and thus inhibit cancer cell apoptosis, which is not conducive to the survival of radiotherapy patients. Studies have shown that highly expressed SRXN1 can inhibit the production of reactive oxygen species (ROS) and reduce apoptosis [37]. Furthermore, previous studies have shown that silencing SRXN1 significantly increases oxidative stress [38]. Therefore, studying the association of SRXN1 expression with the GSVA score of the oxidative stress response may provide insights into the mechanism by which SRXN1 regulates the oxidative stress response in prostate cancer patients after radiotherapy. This study found that there was a significant negative correlation between srxn1 gene expression and the GSVA score of the oxidative stress response in patients. The Wilcoxon rank sum test showed that patients with low SRXN1 expression had higher scores in response to oxidative stress, which further showed that SRXN1 had an antioxidant stress effect.

In conclusion, we have confirmed that radiotherapy can kill tumour cells through DNA damage and induce oxidative stress. However, in prostate cancer, upregulation of the antioxidative stress factor SRXN1 leads to radiotherapy tolerance, which can potentially be used to predict the prognosis of prostate cancer patients after radiotherapy. Due to limited experimental conditions, we plan to knock out or knock down SRXN1 expression in PC3 and DU145 cells and verify whether tumour cell sensitivity to radiotherapy will be increased after inhibiting SRXN1 expression.

## Data Availability

The datasets used and analysed during the current study are presented in the main body of this manuscript.

## Abbreviations

GEO: Gene Expression Omnibus
GSVA: Gene set variation analysis
KEGG: Kyoto Encyclopedia of Genes and Genomes
DAVID: The Database for Annotation, Visualization and Integrated Discovery
lncRNA: Long noncoding RNA
DNA: Deoxyribonucleic acid
TCGA: The Cancer Genome Atlas
MSigDB: The Molecular Signatures Database
PFS: Progression-free interval survival
